# Supplementary material for: Energy Imbalance Gap, Anthropometric Measures, Lifestyle, and Sociodemographic Correlates in Latin American Adults—Results from the ELANS Study
Source: Int J Environ Res Public Health. 2022 Jan 20;19(3):1129. doi: 10.3390/ijerph19031129 (PMC8835004; doi:10.3390/ijerph19031129)
Supplement: Supplementary file 1 [file ijerph-19-01129-s001.zip › ijerph-1444505-supplementary.pdf]

Table S1. Energy Intake Percentiles by sociodemographic characteristics

	3	10	25	50	75	90	97
**Country**							
Argentina	1264.81	1464.32	1690.37	2040.11	2393.58	2771.60	3238.29
Brazil	1114.09	1269.68	1490.29	1782.05	2091.35	2397.64	2815.65
Chile	1095.47	1284.42	1474.05	1705.22	1983.51	2339.49	2788.66
Colombia	1319.51	1512.38	1699.74	1979.53	2295.59	2568.86	3001.80
Costa Rica	1099.39	1255.55	1551.14	1826.03	2171.40	2519.97	2944.01
Ecuador	1371.48	1579.80	1799.30	2110.21	2422.53	2658.31	3019.68
Peru	1290.59	1483.59	1706.35	1983.37	2295.85	2608.55	2955.74
Venezuela	1153.61	1364.11	1540.54	1808.72	2105.01	2451.43	2825.29
ELANS	1170.98	1372.28	1598.88	1893.69	2227.91	2554.17	2950.71
**Gender**							
Male	1360.28	1568.86	1820.00	2114.84	2439.31	2768.72	3163.30
Female	1104.01	1275.04	1480.56	1713.81	1985.75	2250.83	2531.12
**Age group**							
19 to 34	1210.36	1426.01	1676.53	1967.72	2301.80	2642.00	3073.75
35 to 49	1168.81	1377.38	1598.79	1886.73	2226.82	2543.71	2888.95
50 to 65	1122.05	1284.73	1496.86	1743.03	2049.10	2366.75	2746.72
**Socio-economic level**							
Low	1142.16	1347.81	1565.83	1869.68	2226.19	2550.20	2918.13
Middle	1190.32	1390.58	1631.95	1924.55	2230.18	2562.06	2959.14
High	1164.98	1391.58	1638.52	1921.01	2228.22	2557.52	3068.24
**Education level**							
None and basic	1133.20	1345.17	1573.39	1871.54	2233.54	2551.13	2951.46
High school	1220.98	1413.22	1639.19	1930.63	2232.96	2558.37	3001.27
Bachelor´s degree	1186.90	1404.56	1620.37	1905.48	2201.91	2564.93	2824.30
**Ethnicity**							
Caucasian	1141.79	1348.81	1571.73	1860.07	2187.37	2535.84	2902.14
Mestizo	1204.25	1404.59	1641.36	1947.66	2274.80	2581.62	2985.01
Afro-American	1126.43	1369.72	1571.39	1894.09	2226.96	2532.14	2876.12
Indigenous	1241.22	1448.74	1602.44	1835.44	2178.86	2363.22	3026.55
Others	1160.36	1324.08	1532.51	1796.69	2123.77	2505.47	2981.25
**Body mass index**							
Underweight	1184.11	1306.28	1536.08	1826.79	2182.50	2495.29	2806.74
Normal weight	1166.12	1375.10	1625.93	1910.23	2245.33	2555.87	2936.47
Overweight	1171.68	1377.53	1597.00	1899.03	2232.45	2573.45	2967.48
Obese	1169.07	1369.08	1570.61	1859.36	2214.95	2538.71	2945.98

Table S2. Energy Expenditure Percentiles by sociodemographic characteristics

	3	10	25	50	75	90	97
**Country**							
Argentina	1422.96	1521.66	1661.35	1864.42	2126.03	2405.69	2800.57
Brazil	1398.48	1492.01	1612.90	1821.66	2088.25	2382.57	2771.49
Chile	1425.48	1518.38	1677.28	1903.64	2128.70	2402.20	2800.65
Colombia	1387.72	1493.92	1615.53	1818.60	2093.13	2347.55	2718.97
Costa Rica	1412.46	1522.26	1656.12	1878.71	2158.03	2531.90	2828.35
Ecuador	1454.01	1573.36	1724.93	1951.89	2218.84	2550.62	2839.04
Peru	1382.50	1500.67	1627.26	1839.66	2082.21	2419.14	2651.29
Venezuela	1414.23	1524.52	1655.05	1852.15	2125.89	2375.42	2694.00
ELANS	1407.23	1510.54	1645.71	1855.43	2122.43	2411.06	2764.82
**Gender**							
Male	1631.38	1763.48	1916.47	2105.98	2343.68	2632.55	2898.25
Female	1371.90	1454.40	1548.52	1672.44	1828.03	1998.05	2168.86
**Age group**							
19 to 34	1401.04	1514.24	1672.44	1911.89	2198.52	2530.79	2867.50
35 to 49	1463.15	1538.51	1667.67	1865.46	2118.19	2343.17	2688.45
50 to 65	1344.52	1456.81	1580.26	1743.30	1973.75	2222.18	2480.24
**Socio-economic level**							
Low	1394.45	1499.32	1637.04	1828.82	2080.73	2373.10	2709.26
Middle	1422.42	1514.23	1655.05	1881.13	2154.73	2453.91	2805.30
High	1444.72	1538.33	1656.00	1904.83	2168.15	2507.43	2814.78
**Education level**							
None and basic	1400.95	1508.69	1642.15	1835.23	2095.70	2385.12	2716.38
High school	1406.04	1511.28	1654.47	1886.37	2150.73	2451.12	2805.28
Bachelor´s degree	1426.14	1519.20	1635.55	1871.92	2135.41	2428.86	2870.25
**Ethnicity**							
Caucasian	1423.46	1513.50	1641.33	1846.44	2101.52	2370.58	2777.70
Mestizo	1404.94	1512.24	1652.83	1863.65	2128.70	2421.62	2752.22
Afro-American	1382.88	1520.64	1650.19	1859.07	2143.01	2472.32	2807.26
Indigenous	1415.97	1478.83	1607.13	1878.36	2131.63	2345.50	2542.12
Others	1371.69	1490.53	1624.02	1834.77	2140.43	2489.64	2829.49
**Body mass index**							
Underweight	1195.66	1279.58	1381.25	1508.37	1747.64	1982.04	2286.18
Normal weight	1377.17	1443.84	1538.33	1744.94	1972.75	2304.29	2624.51
Overweight	1479.87	1557.96	1655.05	1891.46	2128.46	2378.10	2791.05
Obese	1577.93	1665.89	1770.95	1981.60	2256.63	2514.06	2899.45

Table S3. Energy Imbalance Percentiles by sociodemographic characteristics

	3	10	25	50	75	90	97
**Country**							
Argentina	-707.55	-452.56	-179.60	133.92	468.63	781.11	1182.29
Brazil	-898.59	-608.99	-357.48	-88.81	218.13	473.52	822.60
Chile	-951.56	-650.28	-422.49	-199.87	68.62	329.24	632.50
Colombia	-696.88	-419.17	-156.11	128.06	418.28	714.03	973.43
Costa Rica	-963.90	-670.58	-412.71	-69.97	215.62	508.25	904.23
Ecuador	-673.01	-415.29	-154.02	96.19	415.97	689.84	937.41
Peru	-666.27	-399.32	-145.23	142.63	421.37	665.48	884.25
Venezuela	-805.86	-578.05	-337.77	-51.23	231.85	460.00	733.42
ELANS	-808.50	-536.67	-283.39	12.38	316.31	606.24	894.62
**Gender**							
Male	-938.14	-640.57	-343.34	-11.07	329.32	642.58	976.19
Female	-657.36	-454.57	-237.13	25.04	305.72	583.32	823.50
**Age group**							
19 to 34	-873.00	-545.26	-283.06	42.17	350.69	657.13	959.78
35 to 49	-776.12	-540.86	-279.61	-11.17	305.93	581.49	880.20
50 to 65	-747.98	-515.45	-286.55	-26.42	274.33	539.43	835.38
**Socio-economic level**							
Low	-769.09	-522.29	-275.89	18.97	315.11	620.94	901.80
Middle	-865.15	-550.77	-289.15	5.32	318.07	593.95	917.01
High	-810.09	-570.86	-288.66	2.59	306.72	614.85	847.76
**Education level**							
None and basic	-773.63	-535.87	-282.72	7.24	313.92	601.39	920.86
High school	-861.41	-535.78	-283.45	20.88	323.42	616.53	885.40
Bachelor´s degree	-868.18	-557.55	-286.38	2.61	298.24	605.72	812.49
**Ethnicity**							
Caucasian	-818.57	-565.64	-307.29	-22.11	293.90	605.74	888.96
Mestizo	-747.04	-495.26	-247.54	50.43	363.42	648.14	919.56
Afro-American	-893.45	-529.68	-291.45	12.46	275.29	520.50	890.11
Indigenous	-838.80	-586.41	-302.58	-35.29	397.92	625.28	936.65
Others	-931.42	-598.39	-327.68	-60.36	238.82	504.45	815.95
**Body mass index**							
Underweight	-509.69	-229.93	65.79	252.20	503.05	814.86	1194.90
Normal weight	-627.93	-383.86	-130.08	139.64	416.24	708.01	960.08
Overweight	-785.08	-532.78	-292.66	-11.44	301.03	576.12	893.00
Obese	-983.62	-672.48	-433.44	-151.66	154.10	447.29	762.06
